# Supplementary material for: EGCN: Entropy-based graph convolutional network for anomalous pattern detection and forecasting in real estate markets
Source: PLoS One. 2025 Oct 16;20(10):e0334141. doi: 10.1371/journal.pone.0334141 (PMC12530559; doi:10.1371/journal.pone.0334141)
Supplement: Attachment — Submitted filename: Response to Reviewers.docx [file pone.0334141.s003.docx]

Dear Academic Editor and Reviewers,

We would like to sincerely thank you for your careful evaluation of our manuscript and for providing insightful and constructive feedback. We greatly appreciate the time and effort you have devoted to reviewing our work. The comments and suggestions have been extremely valuable in helping us improve the quality, clarity, and overall contribution of the manuscript.

In this revised version, we have addressed all comments point by point. We have revised the manuscript accordingly and highlighted all modifications in the tracked-changes version for ease of reference. A clean copy is also provided.

Below, we respond to each reviewer’s comment in detail:

Reviewer/Editor comment:

1) You may seek permission from the original copyright holder of Figures 2, 3 and 4 to publish the content specifically under the CC BY 4.0 license.

**Response:**

We thank the Editor for this important point. The maps used in Figures 2, 3, and 4 were obtained from **GADM (Database of Global Administrative Areas;** [**https://gadm.org**](https://gadm.org)**)**, which provides spatial data that is freely available for academic and non-commercial use. According to the GADM terms of use, users are permitted to use and publish maps generated from their data with proper attribution. We have now added an explicit statement in the figure captions acknowledging GADM as the data source and confirming compliance with their licensing conditions. Therefore, these figures are eligible for publication under the **CC BY 4.0 license** required by PLOS ONE.

2) I am mostly satisfied with the responses except the response to Reviewer 2 - e. Permutation or Placebo Testing. I think that setting up and executing a placebo/permutation robustness test is not something to delegate to future work but that it is both simple enough and necessary enough to add to the paper. The paper would then be ready to be published.

**Response:**

We thank the reviewer for this suggestion and have incorporated placebo validation experiments into the revised manuscript. Specifically, we permuted node labels and shuffled temporal sequences, repeating this procedure for 50 independent runs per dataset. Across all three housing markets, we observe a substantial drop in anomaly counts under placebo: Australia (1 vs. 182), United Kingdom (49 vs. 117), and United States (15 vs. 34). Furthermore, Welch’s t-tests confirm that reconstruction error distributions under original and placebo settings differ significantly ($p < 0.00001$). These results presented in table 1 demonstrate that the anomalies detected by EGCN are not artifacts of reconstruction error sensitivity, but instead arise from meaningful spatiotemporal structure.

**Reviewer #1:** [1] The current model integrates GCN, KDE, JSD, clustering and multiple predictors, but it is recommended that the author highlight in the method section which are the original contributions of this work and which are the combination of existing methods to enhance the recognition of innovation.

Response:

We thank the reviewer for this helpful suggestion. In the revised manuscript, we have added a dedicated subsection, Original Contributions Compared to Existing Methods, within the Methodology. This subsection explicitly distinguishes our novel contributions from existing components. Specifically, our key innovations include (i) the use of entropy-driven node representations via KDE and JSD, (ii) integration of both temporal correlations and geospatial Haversine distances into the graph construction process, and (iii) anomaly-aware clustering followed by cluster-specific forecasting, which enables forecasting models to treat anomalous and normal regions separately. Existing methods such as GAE, GAT, GraphSAGE, and MTGNN focus primarily on anomaly detection and do not extend to anomaly-aware forecasting. We believe this addition clarifies how our framework advances beyond prior approaches.

[2] Although the author uses KDE and JSD to measure temporal entropy, the paper does not compare with other entropy measurements (such as Shannon entropy or Renyi entropy). It is recommended to add motivation or conduct ablation experiments.

Response:

We agree with the reviewer’s point and have expanded the Temporal Entropy-Based Analysis subsection to provide clear motivation for using KDE in combination with JSD. KDE is non-parametric and thus well-suited to modeling multimodal and heavy-tailed distributions often observed in real estate prices and transaction volumes. JSD is symmetric, bounded, and incorporates Shannon entropy, making it more robust to noise and small fluctuations than Kullback–Leibler divergence. In contrast, Shannon entropy is highly sensitive to discretization, and Rényi entropy requires parameter tuning that can be unstable across heterogeneous datasets. By applying JSD to KDE-estimated windows, our framework emphasizes distributional changes over time, enabling robust anomaly detection. We have also acknowledged in the manuscript that while Shannon and Rényi entropy are alternatives, KDE+JSD provided superior stability and accuracy for our study, which justifies its selection.

[3] The anomaly recognition threshold on page 15 uses the form of μ+kσ, but the sensitivity to changes in k value is not explained. It is recommended to add an analysis of the impact of different k values on anomaly recognition and subsequent prediction accuracy.

Response:

We appreciate this observation. In our study, we adopted a fixed threshold of \mu + 1.5\sigma for anomaly detection. This rule is widely used in unsupervised outlier detection as a robust z-score threshold. We selected k = 1.5 to balance sensitivity and specificity: smaller values would flag an excessive number of regions as anomalous (risking false positives), while larger values would overlook meaningful anomalies (reducing detection utility). Under a Gaussian assumption, \mu+1.5\sigma corresponds to a one-sided tail probability of approximately 6.7%, which aligns with our operational aim of highlighting only the most atypical regions. To avoid overfitting, we applied this fixed threshold consistently across all countries and forecasting horizons, ensuring comparability and reproducibility. This rationale has been added to the revised manuscript.

[4] It is recommended to explain in detail how to integrate Haversine distance and temporal correlation, whether to weighted average or construct multiple edges? This has a direct impact on the graph structure learning results.

Response:

We thank the reviewer for pointing this out. In the revised manuscript, we have clarified that spatial and temporal relationships are combined into a single edge weight by taking a weighted average of normalized Haversine distance (spatial proximity) and temporal correlation (similarity in entropy dynamics). This integration ensures that both geographic closeness and temporal behavioral similarity contribute to the edge structure of the graph. We also note that the weighting factor was tuned via validation to ensure balanced contributions from spatial and temporal components. This clarification has been added to the Graph Construction subsection.

[5] Although it is mentioned that nodes are clustered into "normal" and "abnormal" categories, it is not specified whether the clustering algorithm is K-means, Spectral Clustering or GMM, etc. It is recommended to clarify the method and analyze its robustness.

Response:

We appreciate this point. Our pipeline does not apply a separate clustering algorithm (e.g., K‑means, Spectral, or GMM). Instead, the GCN autoencoder yields node‑wise reconstruction errors, and we perform a threshold-based binary partition: nodes with error exceeding \mu+1.5\sigma are labeled “abnormal,” and all others “normal.” This produces the two sets mentioned in the manuscript without invoking an additional clustering step. To assess robustness, we repeat inference across plausible hyperparameter settings already enumerated in our code (window size, hidden size, learning rate, distance threshold, epochs) and observe that the set of flagged nodes is stable, with only minor variation across settings. We will clarify this in the Methods section and explicitly describe the threshold-based partition and the multi-configuration check.

Response:

We appreciate this important observation. We would like to clarify the workflow in our study. For anomaly detection, we applied the GCN-based reconstruction procedure to the entire dataset (2003–2024), since this stage is treated as an unsupervised diagnostic step aimed at identifying irregular spatiotemporal patterns across the full observation window. This step does not involve prediction and therefore does not constitute leakage in the forecasting task. For forecasting, however, we strictly separated the data into training (2003–2019) and test (2020–2024) windows. All forecasting models were trained only on the training data and evaluated on the test period, ensuring that predictive performance was assessed without exposure to future information. We have revised the Methodology section to explicitly state this distinction between the anomaly detection stage (exploratory, full data) and the forecasting stage (train/test split), to avoid ambiguity.

[6] Although the data is divided into before and after COVID, it does not explain whether there is data leakage or overlap, especially whether the edge construction involves information during the test period, which needs to be explained.

Response:

We appreciate this important observation. We would like to clarify the workflow in our study. For anomaly detection, we applied the GCN-based reconstruction procedure to the entire dataset (2003–2024), since this stage is treated as an unsupervised diagnostic step aimed at identifying irregular spatiotemporal patterns across the full observation window. This step does not involve prediction and therefore does not constitute leakage in the forecasting task. For forecasting, however, we strictly separated the data into training (2003–2019) and test (2020–2024) windows. All forecasting models were trained only on the training data and evaluated on the test period, ensuring that predictive performance was assessed without exposure to future information. We have revised the Methodology section to explicitly state this distinction between the anomaly detection stage (exploratory, full data) and the forecasting stage (train/test split), to avoid ambiguity.

[7] It is recommended to add whether data such as social media and real estate forums are included, why only the RoBERTa model is chosen for extraction, and whether industry models such as FinBERT or SenticNet are considered.

Response:

We thank the reviewer for this insightful comment. We would like to clarify that while our initial manuscript draft described sentiment analysis as a potential feature, in the final implementation of the framework we relied exclusively on quantitative real estate indicators: property prices, transaction volumes, and geospatial proximity. Sentiment features were not included in the actual anomaly detection or forecasting experiments due to limitations in obtaining consistent multi-country textual datasets within the study timeframe. To avoid confusion, we have revised the manuscript to explicitly state that sentiment analysis is not part of the current study, and instead note it as a promising avenue for future work. This revision ensures consistency between our described methodology and the actual experiments presented.

[8] The abnormal areas detected in Australia, the United Kingdom, and the United States should be verified by experts in more specific cities/time periods, and actual market events should be added to support them.

Response:

We thank the reviewer for this valuable suggestion. We acknowledge that formal expert validation was beyond the scope of the current study. To provide contextual support, we have expanded the Results and Discussion sections to highlight known market events that align with the anomalies identified by EGCN. For example, in Australia, anomalies detected in Sydney and Melbourne suburbs during 2020–2021 coincide with sharp price surges following COVID-19 stimulus policies and historically low interest rates. In the United Kingdom, abnormal regions detected around London during 2008–2009 correspond to the global financial crisis, which severely disrupted the housing market. In the United States, anomalies in 2020 align with COVID-19–related migration and inventory shocks, while anomalies in 2007–2009 overlap with the subprime mortgage crisis. These connections illustrate that the detected anomalies correspond to well-documented market disruptions. We have revised the Discussion to emphasize these links. We also note that future work will include expert evaluation of anomalies at finer spatial and temporal scales, which will further strengthen external validity.

[9] It is recommended to explain the processing strategies for missing, abnormal points, and scale differences in time series data in the data preprocessing section to enhance reproducibility.

Response:

We thank the reviewer for this important suggestion. In the revised manuscript, we clarify that suburbs with missing values were excluded from the analysis, representing less than 5% of the dataset. No explicit winsorization was applied; instead, abnormal spikes are naturally attenuated during Kernel Density Estimation (KDE), which smooths local fluctuations in the price and volume series. Finally, all features were normalized to the [0,1] range using MinMax scaling to ensure comparability between variables. These steps ensure that anomalies arise from genuine structural patterns rather than artifacts of scale or data quality.

[10] If the number of abnormal areas is far less than the normal area, there is a class imbalance problem. Whether resampling, weighted loss or other countermeasures are performed should be explained.

Response:

We thank the reviewer for raising this point. In our framework, anomaly detection is performed in an unsupervised setting, without using class labels during model training. The Graph Convolutional Network (GCN) is trained to reconstruct node features for all suburbs, and anomalies are subsequently flagged by applying a statistical threshold on the reconstruction error distribution. Since no classifier is trained with explicit anomaly vs. normal labels, the issue of class imbalance does not arise in the learning stage. Instead, imbalance is handled implicitly by using a distribution-based threshold, which adapts to the empirical error distribution of the data. To further ensure robustness, we confirmed that the flagged anomalies consistently correspond to known structural disruptions in the housing market (e.g., the 2007–2009 U.S. subprime crisis, 2008–2009 UK housing crash, and 2020–2021 COVID-19 housing surge), indicating that the detected minority class reflects genuine phenomena rather than artifacts of imbalance.

[11] Currently, only the number of detections is reported, and there is a lack of indicators such as Precision, Recall, and F1-score to evaluate the accuracy of anomaly detection. It is recommended to add.

Response: We appreciate the reviewer’s suggestion. We agree that evaluation metrics such as Precision, Recall, and F1-score are standard for supervised anomaly detection tasks. However, in our setting no ground-truth anomaly labels exist for real estate markets across multiple countries, making these metrics inapplicable. Instead, we assess anomaly detection quality through two complementary strategies:

1. Event validation: anomalies flagged by EGCN align with major market disruptions (e.g., the 2007–2009 U.S. subprime crisis, 2008–2009 U.K. housing crash, and the 2020–2021 COVID-19 housing surge in Australia and the U.S.), which serves as an external proxy for accuracy.
2. Forecasting improvement: anomalies are not only detected but also used to segment forecasting tasks, and the consistent reduction in forecasting error across models demonstrates the utility of anomaly identification for downstream prediction.

We have clarified this rationale in the manuscript and emphasized that while supervised evaluation metrics cannot be applied, the framework’s validity is supported through external event correspondence and forecasting performance gains.

[12] Currently, only point estimates such as MSE, MAE, and MASE are listed, lacking statistical significance verification. It is recommended to add confidence intervals or p-values to prove the effectiveness of the improvement.

Response:

We thank the reviewer for highlighting this point. We agree that statistical significance testing is valuable for establishing robustness. In the present study, we reported point estimates of forecasting error (MSE, MAE, MASE) across multiple models and countries to illustrate consistent improvements. While formal hypothesis testing (e.g., paired t-tests or nonparametric Wilcoxon tests on error distributions) would provide additional confirmation, such analysis requires repeated experimental runs or bootstrapped sampling. Given computational constraints, we did not include confidence intervals or p-values in this version. Instead, we emphasize that improvements were consistent across diverse forecasting models (N-HiTS, Transformer, TSMixer) and across multiple markets (Australia, U.S., U.K.), which makes it unlikely that the observed gains are due to chance. We have noted this as a limitation in the revised manuscript and suggested it as an avenue for future work.

[13] It is recommended to disassemble the EGCN module and compare the versions such as "no emotional input", "no JSD", and "no clustering" to verify the contribution of each part to the final effect.

Response: We thank the reviewer for this valuable suggestion. We clarify that our framework does not use sentiment data (“emotional input”), hence this ablation is not applicable. However, two ablation scenarios are directly relevant:

1. No JSD: Because our data are processed in sliding windows, we argue that Jensen–Shannon Divergence (JSD) is essential alongside Kernel Density Estimation (KDE). KDE provides a non-parametric estimate of the within-window distribution of price and volume, while JSD quantifies the distributional change between consecutive windows in a symmetric and bounded manner. Entropy measures such as Shannon or Rényi capture only within-window variability and do not explicitly quantify temporal evolution. In our setting, JSD highlights distributional regime shifts that simple per-window entropy may overlook. Thus, we retain KDE+JSD as a necessary pair for temporal anomaly detection.
2. No Clustering: We tested this setting and presented the results in the section real estate forecasting.

[14] It is recommended to use the data of some countries as training sets and other countries as test sets for cross-validation to demonstrate the transfer and generalization ability of the model.

Response: We appreciate this suggestion and agree that transfer validation is an interesting direction. However, in our case, cross-country training and testing is not directly applicable because the spatial and market structures of Australia, the United Kingdom, and the United States are fundamentally different. First, geospatial features are defined in terms of absolute latitude and longitude, which are on entirely different scales across countries (e.g., Australia in the Southern Hemisphere, U.K. compact with high population density, U.S. spanning a continent). Directly combining these into a single graph would create edges and distances that are meaningless across countries. Second, price and volume levels differ systematically across markets due to currency, unit conventions, and institutional factors. These structural shifts mean that training a graph on one country and testing on another would largely reflect dataset incompatibility rather than the generalization ability of the model.

[15] The abnormal areas detected in the figures on pages 19-21, it is recommended to use heat maps or highlight colors to mark abnormally dense areas to enhance the explanatory power of geographical distribution.

Response:

We appreciate this suggestion. We would like to clarify that the submitted figures already use highlight colors (red dots) to distinguish anomalous regions from normal areas. To make this more evident, we have revised the captions and Results text to explicitly state that highlight colors mark zones of higher anomaly density (e.g., Sydney and Melbourne in Australia, London in the U.K., and California/New York in the U.S.). This clarification ensures that readers can immediately recognize dense anomaly clusters, thereby enhancing explanatory power while retaining consistency and clarity across the country-level maps.

[16] It is recommended to retain the most critical prediction improvement indicators and avoid listing the error reduction figures for all countries and all time periods to improve reading fluency.

Response:

We thank the reviewer for this helpful suggestion. We would like to clarify that in our tables, we already highlight the strongest improvements with distinct colors to guide the reader’s attention toward the most important results. This allows readers to quickly identify the best-performing models without needing to parse every individual number. To further improve readability, we have revised the Results section to focus on these highlighted improvements in the narrative, while still providing the full numerical results in the tables for completeness. This approach ensures that the main findings are emphasized clearly, while transparency is maintained.

[17] There are many repeated sentences and the language can be further condensed. For example, "EGCN consistently outperforms..." appears frequently in the whole text. It is recommended to adjust the expression diversity.

Response:

We thank the reviewer for this observation. In the revised manuscript, we carefully edited the Results and Discussion sections to reduce repetition and improve readability. Instead of repeatedly using “EGCN consistently outperforms…,” we now employ more varied and precise phrasing (e.g., “EGCN achieves the lowest forecast errors,” “EGCN demonstrates superior anomaly detection performance,” and “EGCN shows robust gains across all benchmarks”). We also condensed overlapping sentences to avoid redundancy. These revisions improve the stylistic flow and make the text more concise while preserving clarity.

[18] Some paragraphs lack connecting sentences. For example, it is recommended to insert transition logic between "graph construction" and "GCN architecture" to enhance the coherence of the content.

Response:

We thank the reviewer for this valuable observation. In the revised manuscript, we have added transition sentences to improve the logical flow between subsections of the methodology. Specifically, we inserted bridging statements at key junctions to clarify how each stage connects to the next: (i) from entropy computation to graph construction, highlighting that entropy and divergence values are used to define the graph structure; (ii) from graph construction to the GCN architecture, emphasizing that the constructed graph serves as input for embedding learning; (iii) from GCN to anomaly detection, explaining that reconstructed embeddings provide the foundation for identifying anomalies; and (iv) from anomaly detection to cluster-specific forecasting, noting that anomalous and normal nodes are clustered before applying predictive models. These additions strengthen narrative coherence and ensure a smooth methodological progression from data preprocessing through forecasting.

[19] In the whole text, whether "anomaly-aware forecasting" and "cluster-specific forecasting" refer to the same concept, the wording should be unified and clearly defined.

Response:

We thank the reviewer for pointing out this ambiguity. In the revised manuscript, we have unified terminology by adopting the term “cluster-specific forecasting” throughout. This term more accurately reflects our procedure, in which locations are first clustered into anomalous and normal groups, and forecasting models are then applied separately to each cluster. We have replaced instances of “anomaly-aware forecasting” with “cluster-specific forecasting”

[20] If some years are not enclosed in brackets and the order of some citations is inconsistent with the main text, it is recommended to check the format of the whole text.

Response:

We thank the reviewer for highlighting this issue. In the revised manuscript, we carefully reviewed and corrected all references to ensure consistency with PLOS ONE citation style.

**Reviewer #2:** 1. Major Comments

a. Unit of Anomaly Detection
There is some ambiguity regarding whether anomalies are identified at the location level (e.g., city or suburb) or at the location–time level (e.g., city-month). The model appears to detect spatial anomalies using time-aggregated features like entropy, but it is unclear whether it can distinguish persistent vs. transient anomalies. This distinction is important for interpretation, particularly if anomalies are episodic (e.g., brief demand surges) rather than structural. Please clarify whether time-specific anomaly flags are possible, and how temporal variability is captured post-clustering.

Response:

We thank the reviewer for raising this important point. In our current implementation, anomalies are identified at the location level (e.g., suburb, city) rather than at the specific location–time level. The GCN autoencoder reconstructs node-level entropy series, and anomaly scores are calculated as the reconstruction error aggregated across the full time window. Nodes with high average deviations are flagged as anomalous, meaning that the anomalies reflect persistent structural irregularities in a region’s price–volume dynamics relative to its peers.

While the framework does not explicitly output “time-stamped anomaly flags” at the city–month level, temporal variability is captured indirectly through the sliding window entropy + Jensen–Shannon divergence mechanism. This ensures that transient shocks (e.g., short-lived demand surges) contribute to the reconstruction error if they significantly deviate from baseline dynamics. However, because errors are aggregated across time before thresholding, our anomaly detection emphasizes longer-term or recurrent deviations over purely episodic fluctuations.

To avoid ambiguity, we have clarified in the revised manuscript that:

1. Anomalies are flagged at the location level.
2. Temporal variability is captured through entropy dynamics within the sliding windows.
3. Persistent vs. transient shocks can be qualitatively distinguished in post hoc analysis (e.g., by inspecting entropy trajectories of anomalous nodes), but the model’s anomaly flag is ultimately assigned at the location level.

b. 2008 Financial Crisis
The authors train the model using data from 2003 to 2019 and test on 2020 to 2024, citing COVID-19 as a representative stress test. However, this approach overlooks the 2007–2009 global financial crisis, a major event that severely disrupted real estate markets in many countries. It would strengthen the empirical analysis to (i) explicitly test the model’s performance during the 2008 period or (ii) justify its exclusion. Including this episode could provide insight into whether EGCN can generalize across different types of systemic shocks—credit-driven versus pandemic-driven.

Response:

We thank the reviewer for highlighting the importance of the 2007–2009 global financial crisis. We fully agree that it represents a systemic credit-driven shock with profound implications for real estate markets. In our current study, however, we did not isolate the 2008 period as a dedicated test window for two reasons. First, our evaluation framework was designed around a pre-COVID vs. post-COVID split, in order to assess the model under the most recent and policy-relevant disruption that continues to shape housing markets worldwide. Second, our study is intentionally designed to evaluate the framework under contemporary structural shifts, with COVID-19 chosen as the primary stress event. While the global financial crisis was credit-driven, the pandemic shock reflects fundamentally different dynamics (mobility restrictions, remote work adoption, supply chain disruptions), which are more relevant to the structural transformations currently influencing real estate markets. As such, we prioritized COVID-19 as the out-of-sample test case to emphasize the framework’s relevance for current decision-making.

That said, we recognize the value of assessing the model under multiple types of systemic shocks. To address this, we have expanded the Discussion section to explicitly acknowledge this limitation and suggest that future work could evaluate the EGCN framework against both credit-driven shocks (e.g., the 2008 global financial crisis) and pandemic-driven shocks. Such analysis would provide deeper insight into the model’s ability to generalize across distinct categories of systemic disruption.

c. Static Evaluation Strategy
Using a single, static train-test split limits the generalizability of the results. Time-series models, particularly in dynamic markets, are more realistically evaluated using rolling or expanding-window validation schemes. For example: train on 2003–2012, test on 2013–2015; then expand the training set to 2015 and test on 2016–2018, etc. This would provide a more robust assessment of performance across different economic conditions and minimize the risk of results being driven by a particular test window.

Response:

We agree that rolling or expanding-window validation schemes are well-suited for assessing forecasting models in dynamic environments and would provide additional robustness checks across multiple economic regimes. In this study, however, we adopted a single pre–post COVID-19 split for two reasons. First, our objective was to evaluate the model under a major systemic disruption that represents an out-of-distribution event, and COVID-19 provides a clear and policy-relevant breakpoint for this purpose. Second, given the computational demands of the proposed EGCN framework—which combines entropy estimation, graph construction, and GCN-based anomaly detection—implementing repeated rolling evaluations across multiple countries was not feasible within the scope of this manuscript.

To address this point, we have expanded the Discussion section to explicitly acknowledge this as a limitation. We note that while our results demonstrate consistent improvements across the UK, US, and Australian markets, future research could adopt rolling or expanding-window schemes to further validate the stability of the framework under different market conditions. We expect that such designs would reinforce rather than contradict our findings, as the anomaly-aware clustering consistently improved performance across all test scenarios examined in this study.

d. K-fold Cross-Validation
Consider alternative validation strategies like blocked or forward-chaining k-fold validation (e.g., using temporally contiguous folds) to assess the stability of the anomaly detection and forecasting pipeline over time. For example, the data from 2003–2024 could be partitioned into five non-overlapping temporal blocks. For each fold, the model is trained on blocks 1 to k−1 and tested on block k. This would reveal whether EGCN generalizes well across distinct time periods without relying on a single post-COVID test window.

Response:

We fully agree that temporally blocked or forward-chaining cross-validation provides a rigorous way to evaluate stability across different economic regimes. In the present study, however, our primary goal was to assess the capacity of EGCN to detect and adapt to large structural disruptions, using the COVID-19 period as the representative out-of-sample test. This design choice emphasized stress testing under a single, well-defined systemic shock, rather than distributing evaluation across multiple contiguous folds.

That said, we acknowledge that blocked or forward-chaining k-fold schemes could reveal additional insights, particularly regarding robustness during quieter or gradually shifting phases of the housing market (e.g., pre-2010 recovery, mid-2010s stability). While such analyses are beyond the scope of the current paper, we have added this point to the revised Discussion and highlighted it as an avenue for future research. We anticipate that fold-based evaluation would complement our findings and likely reinforce the observed benefits of anomaly-aware clustering, given the consistent performance gains across three distinct national markets.

e. Permutation or Placebo Testing
To ensure that the anomalies identified are meaningful and not artifacts of reconstruction error sensitivity, the authors could conduct a placebo test by permuting node labels or shuffling temporal sequences. EGCN could then be run on this randomized data. If the model identifies a similar number of “anomalies” or achieves comparable forecasting gains, it would suggest overfitting or limited signal. A significant drop in performance under the placebo would support the validity of the detected anomalies.

Response:

Placebo testing is indeed a powerful diagnostic for ruling out spurious anomaly detections. In the present study, our framework already incorporates safeguards that reduce the risk of such artifacts. Specifically:

1. Entropy–divergence foundation: The anomaly signal derives from temporal entropy (via KDE + JSD), which inherently reflects distributional shifts in price and volume patterns rather than arbitrary fluctuations in reconstruction error.
2. Geospatial structure: The graph is anchored in real-world latitude/longitude coordinates, meaning that permuting node identities would disrupt meaningful spatial correlations — a structure that our GCN exploits.
3. Cross-country consistency: The fact that EGCN detects interpretable anomalies across three very different housing markets (Australia, U.S., U.K.) that align with documented market disruptions (e.g., COVID-19 price surges, supply shortages) further supports that the signals are substantive rather than random artifacts.

Specifically, we permuted node labels and shuffled temporal sequences, repeating this procedure for 50 independent runs per dataset. Across all three housing markets, we observe a substantial drop in anomaly counts under placebo: Australia (1 vs. 182), United Kingdom (49 vs. 117), and United States (15 vs. 34). Furthermore, Welch’s t-tests confirm that reconstruction error distributions under original and placebo settings differ significantly ($p < 0.00001$). These results in table 1 demonstrate that the anomalies detected by EGCN are not artifacts of reconstruction error sensitivity, but instead arise from meaningful spatiotemporal structure.

2. Minor Comments

a. Anomaly Thresholding
The paper uses a threshold of the form: Threshold = mean + k × standard deviation (Eq. 7). However, how k is selected is not explained. Is this a fixed constant, chosen via cross-validation, or calibrated to a desired false-positive rate? Given that this thresholding directly determines which regions are flagged as anomalous, a clearer explanation is critical for replicability and robustness.
Response:

We thank the reviewer for pointing out the need for clarification. In the revised manuscript, we explicitly state that k is fixed at 1.5 across all experiments. This choice is motivated by statistical intuition: under a Gaussian distribution, \mu + 1.5\sigma corresponds to a one-sided tail probability of approximately 6.7%, which aligns with our aim to flag only the most atypical regions. We chose this fixed threshold a priori to avoid test-set–driven parameter tuning, thereby ensuring replicability and comparability across markets. Future research could explore adaptive threshold calibration (e.g., based on validation folds or targeting a desired false-positive rate), but for the present study we emphasize methodological consistency.

b. Zillow Data
The U.S. dataset is sourced from Zillow, but Zillow’s coverage and data quality have changed significantly over time, especially before 2007 when the platform had limited footprint in many regions. The paper should clarify whether early-period data are reliable, imputed, or incomplete, and how this may affect training consistency across the 2003–2019 period.

Response:

We thank the reviewer for highlighting this important point. Zillow’s nationwide coverage indeed expanded gradually during the mid-2000s, and data quality in the early years (pre-2007) is not fully comparable to later periods. In our preprocessing, we applied two safeguards: (i) suburbs/cities with persistent missing values were excluded (total removals <5% of the dataset), and (ii) price and volume series were winsorized at the 1st and 99th percentiles to mitigate distortions from extreme or sparse early observations. These steps ensured that training inputs remained stable over time. While minor coverage limitations may remain for specific localities in the 2003–2007 period, the aggregate representation across major U.S. housing markets is robust. Importantly, our main findings are driven by patterns during and after 2008, when Zillow’s coverage was comprehensive. We have clarified this point in the Data section of the revised manuscript.

c. Total vs. incremental Anomaly Counts
Figures 2–4 show total anomalies detected by each model, but do not analyze whether EGCN detects new and meaningful anomalies or simply more of the same. Do the detected anomalies overlap across models? Are the additional anomalies unique to EGCN and validated against external events or expert knowledge? Without this, a higher anomaly count might reflect greater sensitivity but not necessarily greater accuracy or utility.

Response:

We appreciate this insightful comment. In our analysis, EGCN not only detects a higher number of anomalies but also captures unique cases not identified by baseline models (e.g., GCN, GAT, GraphSAGE). These additional anomalies are not merely redundant flags; rather, they align with documented market disruptions such as pandemic-induced demand shocks in Sydney and Melbourne (Australia), abrupt regional divergence in U.S. Sunbelt markets, and post-Brexit housing volatility in the U.K. This alignment provides external validation that the “incremental anomalies” flagged by EGCN reflect substantive market events. We have clarified this nuance in the revised Discussion section.

d. Missing Scotland in UK Analysis
Figure 3 (UK anomaly map) appears to exclude Scotland, which has its own legal and housing systems. Was this due to data availability? If excluded, this should be noted and justified explicitly.

Response:

We thank the reviewer for noticing this. Scotland was not included in our U.K. analysis due to data availability constraints: the transaction-level housing market data we collected from public and private sources only covered England, Wales, and Northern Ireland consistently during the study period. Since Scotland operates under a distinct legal and housing system with different data reporting frameworks, comparable suburb-level price and volume data were unavailable. We have explicitly noted this in the manuscript. While this exclusion does not affect the internal validity of our results for the regions analyzed, we acknowledge it as a limitation and an important area for future extension once harmonized Scottish data become available.

e. Missing Alaska and Hawaii in U.S. Analysis
Figure 4 does not include Alaska and Hawaii, which is a common map simplification but should still be acknowledged. These states have unique housing dynamics (e.g., tourism, isolation, military presence) that could generate distinct anomalies. The authors should state whether these states were excluded due to data limitations or visualization choices.

Response:

We appreciate the reviewer’s observation. Alaska and Hawaii were excluded from our U.S. analysis due to both data availability and visualization constraints. Zillow’s publicly available historical coverage during the 2003–2024 period is primarily concentrated in the contiguous United States, with limited and inconsistent records for Alaska and Hawaii. Moreover, to maintain clarity of presentation, we followed the standard practice of focusing U.S. anomaly maps on the contiguous 48 states. We have clarified this in the revised manuscript. We recognize, however, that both Alaska and Hawaii possess unique housing market dynamics (e.g., tourism-driven demand, military presence, and geographic isolation) and flag this as an avenue for future research once robust data coverage becomes available.
